# Supplementary material for: Cytoskeletal Organization Correlates to Motility and Invasiveness of Malignant Mesothelioma Cells
Source: Cancers (Basel). 2021 Feb 8;13(4):685. doi: 10.3390/cancers13040685 (PMC7915464; doi:10.3390/cancers13040685)
Supplement: Supplementary file 1 [file cancers-13-00685-s001.pdf]

cancers

MDPI

Supplementary Materials

Cytoskeletal Organization Correlates to Motility and Invasiveness of Malignant Mesothelioma Cells

Maureen Keller, Katarina Reis, Anders Hjerpe, Katalin Dobra and Pontus Aspenström

Table S1. A complete list of the significances of the data presented in Figure 1B–D calculated by one-way ANOVA with Tukey's post hoc analyses.

A			
**Cell Area**			
**Tukey's Multiple Comparisons Test**	**Adjusted *p* Value**	**Summary**	**Significant?**
MeT-5a vs. Mero-25	0.0004	***	Yes
MeT-5a vs. M-14-K	<0.0001	****	Yes
MeT-5a vs. JL-1	0.0537	ns	No
MeT-5a vs. STAV-AB	<0.0001	****	Yes
MeT-5a vs. STAV-FCS	0.025	*	Yes
MeT-5a vs. ZL34	<0.0001	****	Yes
MeT-5a vs. DM-3	<0.0001	****	Yes
Mero-25 vs. M-14-K	0.9787	ns	No
Mero-25 vs. JL-1	0.7245	ns	No
Mero-25 vs. STAV-AB	0.9892	ns	No
Mero-25 vs. STAV-FCS	0.9113	ns	No
Mero-25 vs. ZL34	>0.9999	ns	No
Mero-25 vs. DM-3	<0.0001	****	Yes
M-14-K vs. JL-1	0.1183	ns	No
M-14-K vs. STAV-AB	>0.9999	ns	No
M-14-K vs. STAV-FCS	0.2939	ns	No
M-14-K vs. ZL34	0.9986	ns	No
M-14-K vs. DM-3	<0.0001	****	Yes
JL-1 vs. STAV-AB	0.1349	ns	No
JL-1 vs. STAV-FCS	>0.9999	ns	No
JL-1 vs. ZL34	0.3481	ns	No
JL-1 vs. DM-3	<0.0001	****	Yes
STAV-AB vs. STAV-FCS	0.3325	ns	No
STAV-AB vs. ZL34	0.9997	ns	No
STAV-AB vs. DM-3	<0.0001	****	Yes
STAV-FCS vs. ZL34	0.6317	ns	No
STAV-FCS vs. DM-3	<0.0001	****	Yes
ZL34 vs. DM-3	<0.0001	****	Yes
**B**			
**Cell Shape**			
**Tukey's Multiple Comparisons Test**	**Adjusted *p* Value**	**Summary**	**Significant?**
MeT-5a vs. Mero-25	0.978	ns	No
MeT-5a vs. M-14-K	0.0313	*	Yes
MeT-5a vs. JL-1	<0.0001	****	Yes
MeT-5a vs. STAV-AB	<0.0001	****	Yes
MeT-5a vs. STAV-FCS	<0.0001	****	Yes
MeT-5a vs. ZL34	0.0001	***	Yes
MeT-5a vs. DM-3	<0.0001	****	Yes
Mero-25 vs. M-14-K	0.0023	**	Yes
Mero-25 vs. JL-1	<0.0001	****	Yes

Mero-25 vs. STAV-AB	<0.0001	****	Yes
Mero-25 vs. STAV-FCS	<0.0001	****	Yes
Mero-25 vs. ZL34	<0.0001	****	Yes
Mero-25 vs. DM-3	<0.0001	****	Yes
M-14-K vs. JL-1	0.0002	***	Yes
M-14-K vs. STAV-AB	0.3845	ns	No
M-14-K vs. STAV-FCS	0.2936	ns	No
M-14-K vs. ZL34	0.8794	ns	No
M-14-K vs. DM-3	<0.0001	****	Yes
JL-1 vs. STAV-AB	0.1724	ns	No
JL-1 vs. STAV-FCS	0.3172	ns	No
JL-1 vs. ZL34	0.0156	*	Yes
JL-1 vs. DM-3	<0.0001	****	Yes
STAV-AB vs. STAV-FCS	>0.9999	ns	No
STAV-AB vs. ZL34	0.9896	ns	No
STAV-AB vs. DM-3	<0.0001	****	Yes
STAV-FCS vs. ZL34	0.9652	ns	No
STAV-FCS vs. DM-3	<0.0001	****	Yes
ZL34 vs. DM-3	<0.0001	****	Yes

C

**Cell Aspect Ratio**			
**Tukey's Multiple Comparisons Test**	**Adjusted *p* Value**	**Summary**	**Significant?**
MeT-5a vs. Mero-25	>0.9999	ns	No
MeT-5a vs. M-14-K	0.3107	ns	No
MeT-5a vs. JL-1	0.1358	ns	No
MeT-5a vs. STAV-AB	0.7485	ns	No
MeT-5a vs. STAV-FCS	0.0119	*	Yes
MeT-5a vs. ZL34	0.9928	ns	No
MeT-5a vs. DM-3	0.0001	***	Yes
Mero-25 vs. M-14-K	0.4653	ns	No
Mero-25 vs. JL-1	0.2503	ns	No
Mero-25 vs. STAV-AB	0.8651	ns	No
Mero-25 vs. STAV-FCS	0.0326	*	Yes
Mero-25 vs. ZL34	0.9985	ns	No
Mero-25 vs. DM-3	0.0006	***	Yes
M-14-K vs. JL-1	>0.9999	ns	No
M-14-K vs. STAV-AB	0.9959	ns	No
M-14-K vs. STAV-FCS	0.9227	ns	No
M-14-K vs. ZL34	0.7877	ns	No
M-14-K vs. DM-3	0.2834	ns	No
JL-1 vs. STAV-AB	0.9589	ns	No
JL-1 vs. STAV-FCS	0.9812	ns	No
JL-1 vs. ZL34	0.5398	ns	No
JL-1 vs. DM-3	0.4372	ns	No
STAV-AB vs. STAV-FCS	0.4865	ns	No
STAV-AB vs. ZL34	0.9917	ns	No
STAV-AB vs. DM-3	0.0371	*	Yes
STAV-FCS vs. ZL34	0.0985	ns	No
STAV-FCS vs. DM-3	0.9653	ns	No
ZL34 vs. DM-3	0.002	**	Yes

Table S2. A complete list of the significances of the data presented in Figure 2B, calculated by one-way ANOVA with Tukey's post hoc analysis.

**Focal Adhesion Size**			
**Tukey's Multiple Comparisons Test**	**Adjusted *p* Value**	**Summary**	**Significant?**
Met-5a vs. Mero-25	0.919	ns	No
Met-5a vs. M-14-K	>0.9999	ns	No
Met-5a vs. JL-1	<0.0001	****	Yes
Met-5a vs. STAV-AB	<0.0001	****	Yes
Met-5a vs. STAV-FCS	0.9996	ns	No
Met-5a vs. ZL34	<0.0001	****	Yes
Met-5a vs. DM-3	0.4359	ns	No
Mero-25 vs. M-14-K	0.9963	ns	No
Mero-25 vs. JL-1	<0.0001	****	Yes
Mero-25 vs. STAV-AB	<0.0001	****	Yes
Mero-25 vs. STAV-FCS	0.9991	ns	No
Mero-25 vs. ZL34	<0.0001	****	Yes
Mero-25 vs. DM-3	0.9794	ns	No
M-14-K vs. JL-1	<0.0001	****	Yes
M-14-K vs. STAV-AB	<0.0001	****	Yes
M-14-K vs. STAV-FCS	>0.9999	ns	No
M-14-K vs. ZL34	<0.0001	****	Yes
M-14-K vs. DM-3	0.8173	ns	No
JL-1 vs. STAV-AB	<0.0001	****	Yes
JL-1 vs. STAV-FCS	<0.0001	****	Yes
JL-1 vs. ZL34	<0.0001	****	Yes
JL-1 vs. DM-3	<0.0001	****	Yes
STAV-AB vs. STAV-FCS	<0.0001	****	Yes
STAV-AB vs. ZL34	0.8499	ns	No
STAV-AB vs. DM-3	0.0203	*	Yes
STAV-FCS vs. ZL34	<0.0001	****	Yes
STAV-FCS vs. DM-3	0.8518	ns	No
ZL34 vs. DM-3	<0.0001	****	Yes

Table S3. A complete list of the significances of the data presented in Figure 4B, calculated by one-way ANOVA with Tukey's post hoc analysis.

Tubulin Acetylation			
**Tukey's Multiple Comparisons Test**	**Adjusted *p* Value**	**Summary**	**Significant?**
MeT-5a vs. Mero-25	<0.0001	****	Yes
MeT-5a vs. M-14-K	0.0005	***	Yes
MeT-5a vs. JL-1	<0.0001	****	Yes
MeT-5a vs. STAV-AB	0.6819	ns	No
MeT-5a vs. STAV-FCS	0.9695	ns	No
MeT-5a vs. ZL34	0.0071	**	Yes
MeT-5a vs. DM-3	<0.0001	****	Yes
Mero-25 vs. M-14-K	<0.0001	****	Yes
Mero-25 vs. JL-1	0.9664	ns	No
Mero-25 vs. STAV-AB	0.024	*	Yes
Mero-25 vs. STAV-FCS	<0.0001	****	Yes
Mero-25 vs. ZL34	<0.0001	****	Yes
Mero-25 vs. DM-3	0.2023	ns	No
M-14-K vs. JL-1	<0.0001	****	Yes
M-14-K vs. STAV-AB	<0.0001	****	Yes
M-14-K vs. STAV-FCS	0.0149	*	Yes
M-14-K vs. ZL34	0.9936	ns	No
M-14-K vs. DM-3	<0.0001	****	Yes
JL-1 vs. STAV-AB	0.0008	***	Yes
JL-1 vs. STAV-FCS	<0.0001	****	Yes
JL-1 vs. ZL34	<0.0001	****	Yes
JL-1 vs. DM-3	0.8137	ns	No
STAV-AB vs. STAV-FCS	0.1301	ns	No
STAV-AB vs. ZL34	<0.0001	****	Yes
STAV-AB vs. DM-3	<0.0001	****	Yes
STAV-FCS vs. ZL34	0.1193	ns	No
STAV-FCS vs. DM-3	<0.0001	****	Yes
ZL34 vs. DM-3	<0.0001	****	Yes

Table S4. A complete list of the significances of the data presented in Figure 5B, calculated by one-way ANOVA with Tukey's post hoc analysis.

Vimentin Localization			
**Tukey's Multiple Comparisons Test**	**Adjusted *p* Value**	**Summary**	**Significant?**
MeT-5a vs. Mero-25	0.401	ns	No
MeT-5a vs. M-14-K	0.0012	**	Yes
MeT-5a vs. JL-1	0.9978	ns	No
MeT-5a vs. STAV-AB	>0.9999	ns	No
MeT-5a vs. STAV-FCS	0.0002	***	Yes
MeT-5a vs. ZL34	<0.0001	****	Yes
MeT-5a vs. DM-3	<0.0001	****	Yes
Mero-25 vs. M-14-K	0.3752	ns	No
Mero-25 vs. JL-1	0.8144	ns	No
Mero-25 vs. STAV-AB	0.4524	ns	No
Mero-25 vs. STAV-FCS	0.1527	ns	No
Mero-25 vs. ZL34	<0.0001	****	Yes
Mero-25 vs. DM-3	<0.0001	****	Yes
M-14-K vs. JL-1	0.0106	*	Yes
M-14-K vs. STAV-AB	0.0016	**	Yes
M-14-K vs. STAV-FCS	0.9997	ns	No
M-14-K vs. ZL34	0.012	*	Yes
M-14-K vs. DM-3	<0.0001	****	Yes
JL-1 vs. STAV-AB	0.9991	ns	No
JL-1 vs. STAV-FCS	0.0023	**	Yes
JL-1 vs. ZL34	<0.0001	****	Yes
JL-1 vs. DM-3	<0.0001	****	Yes
STAV-AB vs. STAV-FCS	0.0003	***	Yes
STAV-AB vs. ZL34	<0.0001	****	Yes
STAV-AB vs. DM-3	<0.0001	****	Yes
STAV-FCS vs. ZL34	0.0469	*	Yes
STAV-FCS vs. DM-3	<0.0001	****	Yes
ZL34 vs. DM-3	0.0006	***	Yes

Table S5. A complete list of the significances of the data presented in Figure 7B–E, calculated by one-way ANOVA with Tukey's post hoc analyses.

**A**			
**YAP Nuclear Localization**			
**Tukey's Multiple Comparisons Test**	**Adjusted *p* Value**	**Summary**	**Significant?**
MeT-5a vs. Mero-25	0.9924	ns	No
MeT-5a vs. M-14-K	0.9785	ns	No
MeT-5a vs. JL-1	<0.0001	****	Yes
MeT-5a vs. STAV-AB	>0.9999	ns	No
MeT-5a vs. STAV-FCS	<0.0001	****	Yes
MeT-5a vs. ZL34	0.0009	***	Yes
MeT-5a vs. DM-3	<0.0001	****	Yes
Mero-25 vs. M-14-K	>0.9999	ns	No
Mero-25 vs. JL-1	<0.0001	****	Yes
Mero-25 vs. STAV-AB	0.9994	ns	No
Mero-25 vs. STAV-FCS	<0.0001	****	Yes
Mero-25 vs. ZL34	0.0088	**	Yes
Mero-25 vs. DM-3	<0.0001	****	Yes
M-14-K vs. JL-1	<0.0001	****	Yes
M-14-K vs. STAV-AB	0.9972	ns	No
M-14-K vs. STAV-FCS	<0.0001	****	Yes
M-14-K vs. ZL34	0.0062	**	Yes
M-14-K vs. DM-3	<0.0001	****	Yes
JL-1 vs. STAV-AB	<0.0001	****	Yes
JL-1 vs. STAV-FCS	0.9952	ns	No
JL-1 vs. ZL34	<0.0001	****	Yes
JL-1 vs. DM-3	0.6521	ns	No
STAV-AB vs. STAV-FCS	<0.0001	****	Yes
STAV-AB vs. ZL34	0.0025	**	Yes
STAV-AB vs. DM-3	<0.0001	****	Yes
STAV-FCS vs. ZL34	<0.0001	****	Yes
STAV-FCS vs. DM-3	0.2636	ns	No
ZL34 vs. DM-3	<0.0001	****	Yes
**B**			
**Nuclear Shape**			
**Tukey's Multiple Comparisons Test**	**Adjusted *p* Value**	**Summary**	**Significant?**
MeT-5a vs. Mero-25	0.0007	***	Yes
MeT-5a vs. M-14-K	>0.9999	ns	No
MeT-5a vs. JL-1	0.9998	ns	No
MeT-5a vs. STAV-AB	0.0846	ns	No
MeT-5a vs. STAV-FCS	0.0143	*	Yes
MeT-5a vs. ZL34	<0.0001	****	Yes
MeT-5a vs. DM-3	0.0003	***	Yes
Mero-25 vs. M-14-K	0.0007	***	Yes
Mero-25 vs. JL-1	0.0001	***	Yes
Mero-25 vs. STAV-AB	0.7688	ns	No
Mero-25 vs. STAV-FCS	0.9995	ns	No
Mero-25 vs. ZL34	<0.0001	****	Yes
Mero-25 vs. DM-3	<0.0001	****	Yes
M-14-K vs. JL-1	0.996	ns	No
M-14-K vs. STAV-AB	0.1056	ns	No
M-14-K vs. STAV-FCS	0.0177	*	Yes
M-14-K vs. ZL34	<0.0001	****	Yes
M-14-K vs. DM-3	<0.0001	****	Yes
JL-1 vs. STAV-AB	0.0249	*	Yes
JL-1 vs. STAV-FCS	0.0038	**	Yes
JL-1 vs. ZL34	<0.0001	****	Yes
JL-1 vs. DM-3	0.0024	**	Yes
STAV-AB vs. STAV-FCS	0.9847	ns	No

STAV-AB vs. ZL34	<0.0001	****	Yes
STAV-AB vs. DM-3	<0.0001	****	Yes
STAV-FCS vs. ZL34	<0.0001	****	Yes
STAV-FCS vs. DM-3	<0.0001	****	Yes
ZL34 vs. DM-3	0.001	***	Yes

C

**Nuclear Aspect Ratio**			
**Tukey's Multiple Comparisons Test**	**Adjusted *p* Value**	**Summary**	**Significant?**
MeT-5a vs. Mero-25	0.0003	***	Yes
MeT-5a vs. M-14-K	0.9418	ns	No
MeT-5a vs. JL-1	0.5708	ns	No
MeT-5a vs. STAV-AB	0.8904	ns	No
MeT-5a vs. STAV-FCS	0.7795	ns	No
MeT-5a vs. ZL34	<0.0001	****	Yes
MeT-5a vs. DM-3	0.0209	*	Yes
Mero-25 vs. M-14-K	0.0105	*	Yes
Mero-25 vs. JL-1	<0.0001	****	Yes
Mero-25 vs. STAV-AB	0.0307	*	Yes
Mero-25 vs. STAV-FCS	0.1907	ns	No
Mero-25 vs. ZL34	<0.0001	****	Yes
Mero-25 vs. DM-3	<0.0001	****	Yes
M-14-K vs. JL-1	0.0393	*	Yes
M-14-K vs. STAV-AB	>0.9999	ns	No
M-14-K vs. STAV-FCS	0.9991	ns	No
M-14-K vs. ZL34	<0.0001	****	Yes
M-14-K vs. DM-3	0.0001	***	Yes
JL-1 vs. STAV-AB	0.0308	*	Yes
JL-1 vs. STAV-FCS	0.0283	*	Yes
JL-1 vs. ZL34	0.0036	**	Yes
JL-1 vs. DM-3	0.805	ns	No
STAV-AB vs. STAV-FCS	>0.9999	ns	No
STAV-AB vs. ZL34	<0.0001	****	Yes
STAV-AB vs. DM-3	0.0001	***	Yes
STAV-FCS vs. ZL34	<0.0001	****	Yes
STAV-FCS vs. DM-3	0.0002	***	Yes
ZL34 vs. DM-3	0.4475	ns	No

D

**Nuclear Perimeter**			
**Tukey's Multiple Comparisons Test**	**Adjusted *p* Value**	**Summary**	**Significant?**
MeT-5a vs. Mero-25	0.0049	**	Yes
MeT-5a vs. M-14-K	<0.0001	****	Yes
MeT-5a vs. JL-1	0.2205	ns	No
MeT-5a vs. STAV-AB	<0.0001	****	Yes
MeT-5a vs. STAV-FCS	0.0009	***	Yes
MeT-5a vs. ZL34	0.0453	*	Yes
MeT-5a vs. DM-3	<0.0001	****	Yes
Mero-25 vs. M-14-K	0.9458	ns	No
Mero-25 vs. JL-1	0.8847	ns	No
Mero-25 vs. STAV-AB	<0.0001	****	Yes
Mero-25 vs. STAV-FCS	0.9984	ns	No
Mero-25 vs. ZL34	<0.0001	****	Yes
Mero-25 vs. DM-3	<0.0001	****	Yes
M-14-K vs. JL-1	0.1418	ns	No
M-14-K vs. STAV-AB	0.0002	***	Yes
M-14-K vs. STAV-FCS	>0.9999	ns	No
M-14-K vs. ZL34	<0.0001	****	Yes
M-14-K vs. DM-3	<0.0001	****	Yes
JL-1 vs. STAV-AB	<0.0001	****	Yes
JL-1 vs. STAV-FCS	0.5463	ns	No

JL-1 vs. ZL34	<0.0001	****	Yes
JL-1 vs. DM-3	<0.0001	****	Yes
STAV-AB vs. STAV-FCS	0.0008	***	Yes
STAV-AB vs. ZL34	<0.0001	****	Yes
STAV-AB vs. DM-3	<0.0001	****	Yes
STAV-FCS vs. ZL34	<0.0001	****	Yes
STAV-FCS vs. DM-3	<0.0001	****	Yes
ZL34 vs. DM-3	<0.0001	****	Yes
	**E**		
**Nuclear Area**			
**Tukey's Multiple Comparisons Test**	**Adjusted *p* Value**	**Summary**	**Significant?**
MetT-5a vs. Mero-25	0.2874	ns	No
MetT-5a vs. M-14-K	<0.0001	****	Yes
MetT-5a vs. JL-1	0.5501	ns	No
MetT-5a vs. STAV-AB	<0.0001	****	Yes
MetT-5a vs. STAV-FCS	0.0503	ns	No
MetT-5a vs. ZL34	>0.9999	ns	No
MetT-5a vs. DM-3	<0.0001	****	Yes
Mero-25 vs. M-14-K	0.4344	ns	No
Mero-25 vs. JL-1	0.9998	ns	No
Mero-25 vs. STAV-AB	<0.0001	****	Yes
Mero-25 vs. STAV-FCS	0.9911	ns	No
Mero-25 vs. ZL34	0.1457	ns	No
Mero-25 vs. DM-3	<0.0001	****	Yes
M-14-K vs. JL-1	0.1489	ns	No
M-14-K vs. STAV-AB	0.0138	*	Yes
M-14-K vs. STAV-FCS	0.969	ns	No
M-14-K vs. ZL34	<0.0001	****	Yes
M-14-K vs. DM-3	<0.0001	****	Yes
JL-1 vs. STAV-AB	<0.0001	****	Yes
JL-1 vs. STAV-FCS	0.9068	ns	No
JL-1 vs. ZL34	0.3477	ns	No
JL-1 vs. DM-3	<0.0001	****	Yes
STAV-AB vs. STAV-FCS	0.0023	**	Yes
STAV-AB vs. ZL34	<0.0001	****	Yes
STAV-AB vs. DM-3	<0.0001	****	Yes
STAV-FCS vs. ZL34	0.0182	*	Yes
STAV-FCS vs. DM-3	<0.0001	****	Yes
ZL34 vs. DM-3	<0.0001	****	Yes

Table S6. Characteristics of the cell lines used in the study.

Cell Line	Origin	Phenotype	Original Reference
MeT-5a	Pleural fluids from non-cancerous male donor. Transfected with pRSV-T plasmid (SV40 early T antigen)	epithelial	[1]
Mero-25	Autopsy from 60 year-old male donor. Asbestos-related	epithelioid	[2]
STAV-AB	Pleural fluid from 59 year-old male donor.	epithelioid	[3]
STAV-FCS	same as STAV-AB	biphasic, fibroblastic	[3]
M-14-K	Fibrous pleural tumor from male smoker. Asbestos-related	epithelioid, biphasic	[4]
ZL34	Fibrous tumor of pleural cavity from 53 year-old male donor. Asbes-tos-related	biphasic	[5]
JL-1	Biopsy from a 54 year-old male donor. Asbestos-related.	epithelioid, biphasic	[6]
DM-3	Biopsy from a 62 year-old male donor. Asbestos-related.	sarcomatoid	[6]

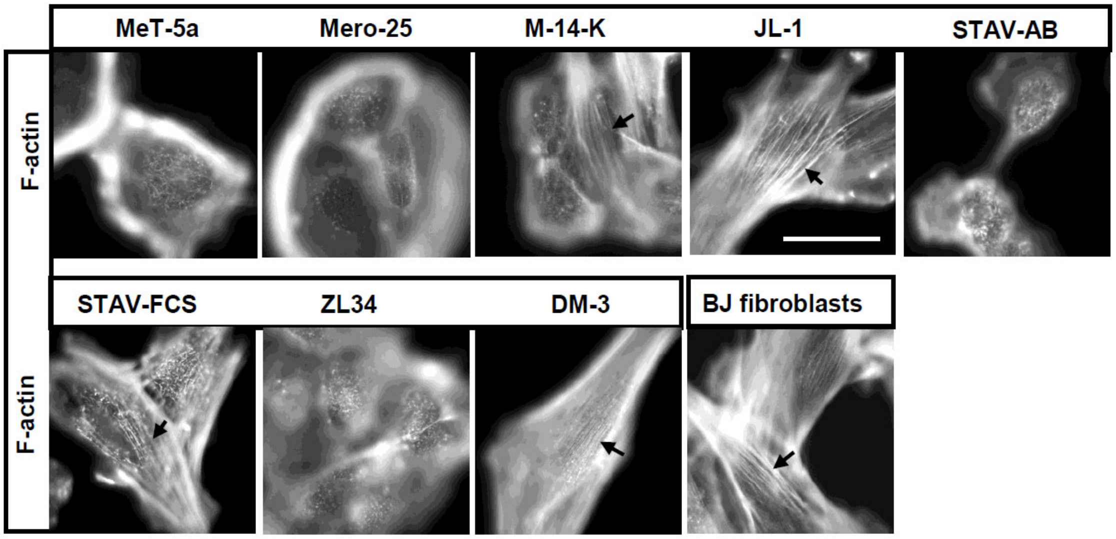

Figure S1. Visualization of actin caps.

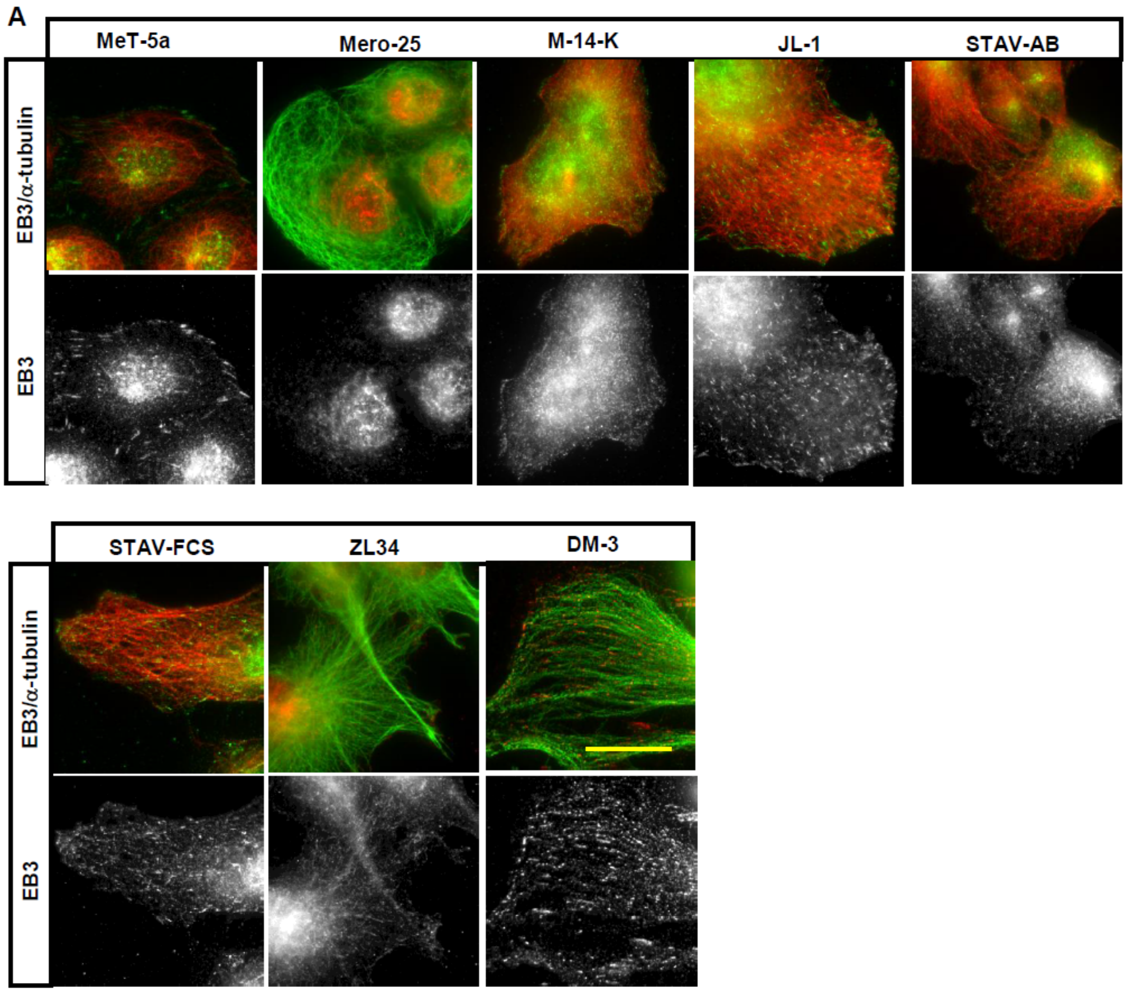

Figure S2. Visualization of microtubule ends.

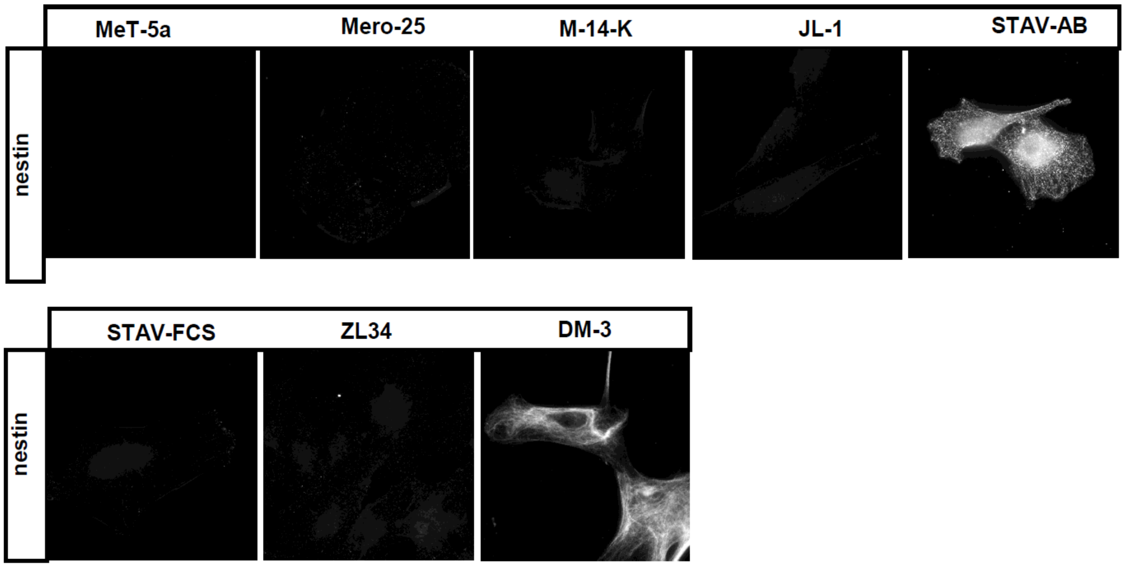

Figure S3. Visualization of nestin intermediate filaments.

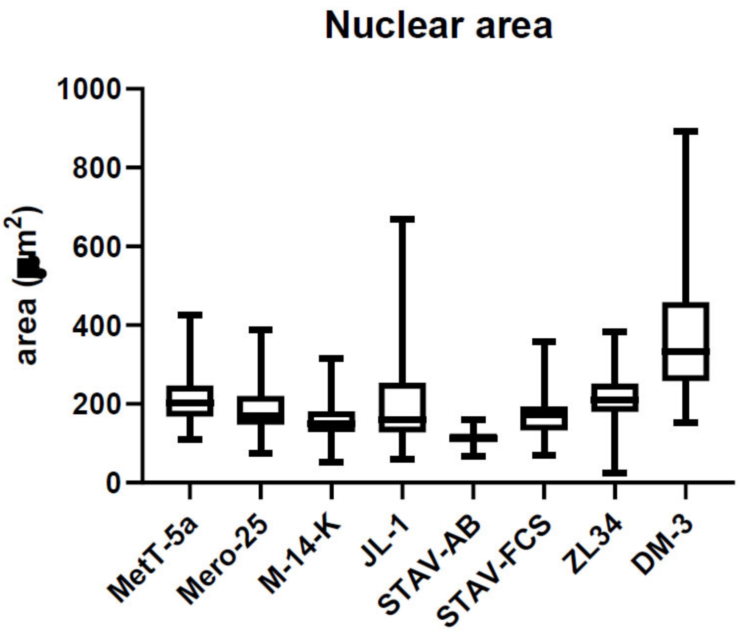

Figure S4. Nuclear area.

Reference

1. Ke. Y.; Reddel, R.R.; Gerwin, B.I.; Reddel, H.K.; Somers, A.N.; McMenamin, M.G.; LaVeck, M.A.; Stahel, R.A.; Lechner, J.F.; Harris, C.C. Establishment of a human in vitro mesothelial cell model system for investigating mechanisms of asbestos-induced mesothelioma. *Am. J. Pathol.* **1989**, *134*, 979–991.
2. Versnel, M.A.; Hoogsteden, H.C.; Hagemeijer, A.; Bouts, M.J.; van der Kwast, T.H.; Delahaye, M.; Schaart, G.; Ramaekers, F.C. Characterization of three human malignant mesothelioma cell lines. *Cancer Genet. Cytogenet.* **1989**, *42*, 115–128.
3. Klominek, J.; Robért, K.H.; Hjerpe, A.; Wickström, B.; Gahrton, G. Serum-dependent growth patterns of two, newly established human mesothelioma cell lines. *Cancer Res.* **1989**, *49*, 6118–6122.
4. Pelin-Enlund, K.; Husgafvel-Pursiainen, K.; Tammilehto, L.; Klockars, M.; Jantunen, K.; Gerwin, B.I.; Harris, C.C.; Tuomi. T.; Vanhala, E.; Mattson, K.; et al. Asbestos-related malignant mesothelioma: Growth, cytology, tumorigenicity and consistent chromosome findings in cell lines from five patients. *Carcinogenesis* **1990**, *11*, 673–681.
5. Schmitter, D.; Lauber, B.; Fagg, B.; Stahel, R.A. Hematopoietic growth factors secreted by seven human pleural mesothelioma cell lines: Interleukin-6 production as a common feature. *Int. J. Cancer* **1992**, *51*, 296–301.
6. Philippeaux, M.M.; Pache, J.C.; Dahoun, S.; Barnet, M.; Robert, J.H.; Mauël, J.; Spiliopoulos, A. Establishment of permanent cell lines purified from human mesothelioma: Morphological aspects, new marker expression and karyotypic analysis. *Histochem. Cell Biol.* **2004**, *122*, 249–260.
